# Developing Sugar‐Free Chewing Gum With Stevia as an Aspartame Alternative

**DOI:** 10.1002/fsn3.70296

**Published:** 2025-05-15

**Authors:** Ayse Aykut, Celale Kirkin

**Affiliations:** ^1^ Department of Food Engineering Faculty of Chemical and Metallurgical Engineering, Istanbul Technical University Istanbul Türkiye

**Keywords:** aspartame, chewing gum, sorbitol, stevia, xylitol

## Abstract

Chewing gum is a commonly used food product, and sugar‐free chewing gum consumption is also frequent. Although artificial sweeteners, such as aspartame, are used in food recipes to replace sugar, they can be associated with health problems. Thus, it is necessary to develop products with natural sweeteners and polyols as sugar substitutes. This study aimed to investigate the use of stevia and xylitol in the production of chewing gum. Four different recipes for chewing gums (sorbitol + aspartame, sorbitol + stevia, sorbitol + xylitol + aspartame, and sorbitol + xylitol + stevia) were created by keeping the amounts of the ingredients other than the sweeteners and polyols constant. The differences in the color, texture, and sensory properties of the chewing gum samples were evaluated. Accelerated shelf‐life test (AST) was also employed to evaluate the stability of the samples during storage. The substitution of sorbitol with xylitol caused color changes (decreased L*, a*, and *h*° values) and decreased the hardness, springiness, and overall likeliness of the samples; however, the use of stevia did not affect the color, texture, and sensory properties of the samples compared to the aspartame‐containing samples. Thus, the findings of this study suggest that stevia can be used in chewing gum production as an alternative to aspartame without causing any adverse effects on the color, texture, and sensory properties.

## Graphical abstract

Stevia exhibited no effects on the color, texture, and sensory properties of the chewing gums. However, xylitol caused changes in the color and texture values, and it decreased the sensory scores. Stevia has a high potential to be used in chewing gum production.

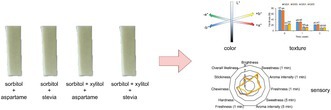

## 1 Introduction

Chewing gum is a confectionary product commonly enjoyed by consumers of a wide range of ages. There have been studies that claim that chewing gum can improve memory (Wilkinson et al. 2002), prevent stress (A. P. Smith 2009), and promote positive mood, attention, and alertness (A. Smith 2010). Additionally, it was stated that sugar‐free chewing gum can help salivary flow, remove substrate, raise plaque pH, and remineralization (Imfeld 1999).

The interest of consumers toward sugar‐free and natural products is increasing due to the fact that health problems, such as diabetes, dental problems, cardiovascular disease, and cancer, can arise with the increase in sugar consumption (Ashwath Kumar and Sudha 2021; Phipps et al. 2020). As a consequence, the development of food products with sweeteners to replace sugar by the industry has been increasing (van der Sman et al. 2022).

The use of sweeteners in food products has several advantages, such as enhancing flavor and being cheaper than sugar (Tandel 2011). They can be categorized as synthetic and natural sweeteners (Schiatti‐Sisó et al. 2023; Tandel 2011). Aspartame is a commonly used synthetic sweetener in chewing gums and other products. It is approximately 180 times sweeter than sucrose (Aravinth et al. 2018; Kroger et al. 2006). However, artificial sweeteners can be associated with some health problems, and their consumption should be limited (Chattopadhyay et al. 2014; More et al. 2021). Thus, it is desired to replace artificial sweeteners with natural alternatives.

Polyols and stevia are widely used natural sweeteners (Schiatti‐Sisó et al. 2023; Tandel 2011). Polyols can be used in food products as bulk sweeteners instead of artificial sugar substitutes. Polyols, unlike sugar, do not cause tooth decay, but their high consumption can cause digestive problems (Chattopadhyay et al. 2014). Sorbitol has been widely used as a bulk sweetener in chewing gum production, but it has been suggested that the addition of xylitol in chewing gum formulations can be more effective in controlling dental plaque formation and acid production than the use of sorbitol alone (Burt 2006; Söderling and Pienihäkkinen 2022; Topitsoglou et al. 1983).

In addition, stevia has the potential to replace sugar and artificial sweeteners, as it is regarded as a safe sweetener being approximately 100–300 times sweeter than sucrose (Goyal et al. 2010; Schiatti‐Sisó et al. 2023). Stevia sweetener is generally obtained as a white powdered extract of *Stevia rebaudiana* leaves, and it is mainly composed of steviol glucosides, which are responsible for the sweet taste (Goyal et al. 2010; Samuel et al. 2018; Singh and Rao 2005). It also has a good composition of bioactive compounds (Schiatti‐Sisó et al. 2023).

Furthermore, the consumption of chewing gums with stevia and xylitol was effective in the inhibition of salivary *Streptococcus mutans* (Shinde and Winnier 2020a). It was also reported that both stevia‐ and xylitol‐containing chewing gums increased salivary flow rate and pH, but the stevia‐containing samples demonstrated a bitter aftertaste (Shinde and Winnier 2020b). In addition, several studies have investigated the effects of using stevia as a sweetener in different food products (Ahmed et al. 2023; Karakütük et al. 2023; Mayangsari et al. 2019; Narayanan et al. 2014; Ozdemir et al. 2015; Pourmohammadi et al. 2017). However, to the best of our knowledge, no studies have evaluated the effects of stevia on the production of chewing gum to replace aspartame. This study sought to determine how the color, texture, and sensory characteristics of chewing gums were affected by the use of stevia and sorbitol and xylitol polyols in place of the commonly used artificial sweetener aspartame. Additionally, an accelerated shelf‐life test (AST) was used to assess the stability of the chewing gums during storage.

## 2 Material and Methods

### 2.1 Preparation of the Samples

The chewing gum samples were prepared in three replications using four different formulations as given in Table 1. All materials used in the recipes were obtained from Perfetti Van Melle (İstanbul, Türkiye). The amounts of the gum base, glycerin, and mint flavor were kept constant in all the recipes. The amounts of stevia and aspartame were determined according to their potency (Tandel 2011), and the total amounts of the polyols and sweeteners were the same in all the recipes. The chewing gum batter was prepared by mixing all the ingredients, and the samples were prepared in 1.5 cm × 4 cm × 0.6 cm dimensions. All samples were packaged in individual plastic (polyethylene) bags and stored at 40°C with relative humidity of 50% and 80% (with 12 cycles) for 2 weeks.

**TABLE 1:** Formulations of the chewing gum samples.

Ingredient (g/100 g)	Samples			
	GSA	GSS	GXA	GXS
Gum base	30	30	30	30
Glycerin	14	14	14	14
Mint flavor	1	1	1	1
Sorbitol	54.7	54.8	27.3	27.4
Xylitol	0	0	27.4	27.4
Aspartame	0.3	0	0.3	0
Stevia	0	0.2	0	0.2

Abbreviations: GSA, chewing gum samples prepared with sorbitol and aspartame; GSS, chewing gum samples with sorbitol and stevia; GXA, chewing gum samples with sorbitol, xylitol, and aspartame; GXS, chewing gum samples with sorbitol, xylitol, and stevia.

### 2.2 Color Measurement

The CIE L* (lightness), a* (redness when positive, greenness when negative), and b* (yellowness when positive, blueness when negative) values of the samples were measured using a colorimeter (CR‐400; Konica Minolta, Japan). The chroma (C*) and hue angle (*h*°) were calculated using the following equations.

$$C^{*}=\sqrt{a^{*2}+b^{*2}}$$

$$h^{{}^{\circ}}=180+\tan^{-1}\left(\frac{b^{*}}{a^{*}}\right)$$

### 2.3 Texture Profile Analysis

The hardness, cohesiveness, springiness, chewiness, and adhesiveness values of the samples were measured using a texture profile analyzer (TA Plus, Lloyd Instruments, UK) coupled with a 3‐mm cylindric probe and 250‐N load cell. The test speed, penetration depth, and trigger force were 2 mm/s, 25%, and 0.1 N, respectively.

### 2.4 Sensory Analysis

Sensory evaluation of the samples was conducted with seven trained panelists who had professional expertise in and routinely practiced chewing gum evaluation. The samples were coded with 3‐digit random numbers, and the panelists were asked to chew the samples for 5 min. They cleansed their mouth with water and dried bread between the samples. Ground coffee was used to neutralize the smell. The brightness, sweetness, aroma intensity, freshness, hardness, chewiness, and stickiness were evaluated using a 9‐point hedonic scale (1: extremely weak, 9: extremely strong). The sweetness, aroma intensity, and freshness were evaluated after 1 and 5 min of chewing. In addition, overall likeliness was evaluated using a 9‐point scale (1: dislike extremely, 9: like extremely).

### 2.5 Statistical Analysis

Evaluation of the data was realized by the analysis of variance, Tukey's multiple comparisons, and Pearson correlation tests using Minitab 16 (Minitab, USA) statistical software.

## 3 Results and Discussion

### 3.1 Color

The L* and a* values of the samples decreased, whereas the b*, C*, and *h*° values increased with time (*p* < 0.05), as shown in Table 2. The replacement of aspartame with stevia did not change the L*, a*, b*, or C* values of the samples (*p* > 0.05). To our knowledge, this is the first study to investigate the effect of replacing aspartame with stevia in sugar‐free chewing gums. However, the use of stevia and its effects on the color of different food products has been reported by several researchers. For instance, using stevia for substituting sugar did not cause a significant color change in functional gummy candies (Roudbari et al. 2024). On the contrary, it has been stated that stevia can play a role in the color of food products even though it is mainly used as a sweetener (Schiatti‐Sisó et al. 2023). Also, the substitution of sucrose with stevia decreased the L*, a*, and b* values of biscuits, which were also lower than those of the samples containing maltitol (Garcia‐Serna et al. 2014). In another study, the concentration of stevia affected the color values of ice cream (Gençdağ et al. 2021). Moreover, the addition of stevia increased a* and b* values of sherbets compared with those of the samples containing sucrose (Karakütük et al. 2023). It should be noted that the stevia‐related changes reported in the aforementioned studies were due to the replacement of sucrose, and all samples used in the present study were sugar‐free.

**TABLE 2:** Color values of the chewing gum samples during storage.

Parameter	Time (week)	Sample			
		GSA	GSS	GXA	GXS
L*	0	87.22^aA^	86.75^aA^	83.78^aA^	85.67^aA^
	1	87.70^aA^	88.34^aA^	82.26^bA^	82.46^bAB^
	2	88.13^aA^	86.66^aA^	78.81^bB^	80.66^bB^
a*	0	−1.99^aA^	−2.19^aA^	−2.14^aA^	−1.72^aA^
	1	−2.41^aA^	−2.58^aA^	−2.61^aAB^	−2.59^aB^
	2	−2.36^aA^	−2.18^aA^	−3.05^bB^	−2.68^abB^
b*	0	6.86^abA^	7.38^aA^	6.45^abA^	5.49^bB^
	1	7.47^aA^	7.54^aA^	7.83^aA^	8.34^aA^
	2	7.60^bA^	7.52^bA^	7.72^abA^	8.31^aA^
*h*°	0	178.71^bA^	178.72^abAB^	178.75^aB^	178.73^abA^
	1	178.74^aA^	178.76^aA^	178.75^aB^	178.74^aA^
	2	178.73^bA^	178.71^bB^	178.81^aA^	178.74^bA^
C*	0	7.14^abA^	7.70^aA^	6.79^abA^	5.75^bB^
	1	7.84^aA^	7.97^aA^	8.26^aA^	8.44^aA^
	2	7.96^abA^	7.84^bA^	8.30^abA^	8.73^aA^

*Note:* Data are the mean of three replications. Values labeled with different lowercase superscript letters in a row and values labeled with different uppercase letters in a column are different (*p* < 0.05).

Abbreviations: GSA, chewing gum samples prepared with sorbitol and aspartame; GSS, chewing gum samples with sorbitol and stevia; GXA, chewing gum samples with sorbitol, xylitol, and aspartame; GXS, chewing gum samples with sorbitol, xylitol, and stevia.

Besides, the samples containing xylitol had lower L* and a* values but higher *h*° values (*p* < 0.05). The substitution of sorbitol with xylitol did not affect the b* and C* values of the samples (*p* > 0.05).

In accordance with our findings, a time‐dependent decrease was also observed in the L* and a* values of chewing gums with lyophilized Jamun extract filled microgel (Sharma et al. 2024). It should be noted that no colorant was used in this study; thus, the changes in the color values can be attributed to the use of sweeteners. However, the color change was only due to the storage time and use of xylitol, not stevia.

### 3.2 Texture

**FIGURE 1:**
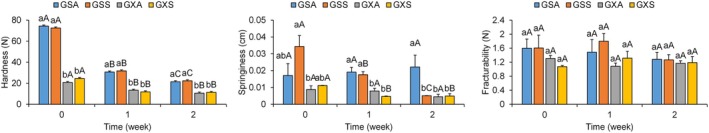
Texture of the chewing gum samples during storage. Data are the mean of three replications. Error bars represent standard deviations. The lowercase letters indicate differences between the samples within a week, whereas the uppercase letters indicate the changes during storage. GSA, chewing gum samples prepared with sorbitol and aspartame; GSS, chewing gum samples with sorbitol and stevia; GXA, chewing gum samples with sorbitol, xylitol, and aspartame; GXS, chewing gum samples with sorbitol, xylitol, and stevia.

The texture properties of the chewing gum samples as affected by the addition of xylitol and stevia during storage are demonstrated in Figure 1. The replacement of aspartame with stevia did not change the hardness, springiness, and fracturability of the samples (*p* > 0.05). On the contrary, the substitution of sorbitol with xylitol resulted in lower hardness and springiness (*p* < 0.05). The hardness of xylitol‐containing samples was lower than that of the other samples (*p* < 0.05). This could be related to the fact that sorbitol has a higher potential to lead to a firmer chewing gum than xylitol (Hartel et al. 2018). Besides, hardness has been claimed to be one of the most important properties that affect the sensory perception of chewing gums (Palabiyik et al. 2020). Although low hardness values are favorable for consumer acceptance, they can increase structural deformation risk (Palabiyik et al. 2020). On the other hand, the hardness values of the samples were greater than those previously reported (Gargouri et al. 2024; Mohammadi et al. 2019).

In this study, the springiness values of the samples were low; however, similar springiness values were also reported previously (Saberi et al. 2018). It has been stressed in some studies that low springiness values were associated with ease of chewing (Saberi et al. 2018; Santos et al. 2014). High springiness values are associated with high chewiness, which is undesirable in chewing gums, as high chewiness values mean more energy for chewing (Santos et al. 2014).

Moreover, low fracturability values have been favored because they are related to brittleness (Gargouri et al. 2024; Santos et al. 2014). The fracturability values of the chewing gum samples ranged between 1.1 and 1.8 N in this study, which can be regarded as desirable.

It should be noted that only unchewed samples were used in this study, and the results of a texture profile analysis could be different from those of a chewed gum sample (Palabiyik et al. 2018).

### 3.3 Sensory Evaluation

**FIGURE 2:**
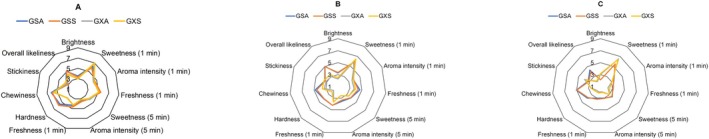
Sensory properties of the chewing gum samples (A) at the beginning, (B) after 1 week, and (C) after 2 weeks. Data are the mean of three replications. GSA, chewing gum samples prepared with sorbitol and aspartame; GSS, chewing gum samples with sorbitol and stevia; GXA, chewing gum samples with sorbitol, xylitol, and aspartame; GXS, chewing gum samples with sorbitol, xylitol, and stevia.

The replacement of aspartame with stevia did not affect the sensory properties of the chewing gum samples (*p* > 0.05), as given in Figure 2. This suggests that stevia can replace aspartame in chewing gum formulations without compromising sensory perception. Similar findings were also obtained in some studies conducted with food products other than chewing gums. For example, yogurt containing aspartame was more preferred than the yogurt samples with sucrose, sucralose, and tagatose, although it was not significantly different from that containing stevia (Medel‐Marabolí et al. 2024). In addition, cocoa drink samples containing an aspartame/acesulfame K mixture and stevia extract were preferred over the samples containing different sugars and sweeteners (Belščak‐Cvitanović et al. 2010).

The freshness (1–5 min), hardness, chewiness, and stickiness of the samples were perceived less strongly in the samples with xylitol (*p* < 0.05). The overall likeliness of the samples prepared with xylitol was also lower than that of the samples prepared without xylitol (*p* < 0.05). On the other hand, the sweetness of the samples was stronger in the samples with xylitol in the first minute of the chewing process (*p* < 0.05), but the difference was lost after chewing the samples for 5 min (*p* > 0.05). The aroma intensity of the samples with xylitol after 5 min of chewing was weaker (*p* < 0.05). Besides, the brightness, sweetness, aroma intensity, freshness, hardness, and overall likeliness of the samples decreased during storage (*p* < 0.05). Overall, it can be concluded that stevia is a good alternative to aspartame as it helps maintain the sensory quality, but the substitution of sorbitol with xylitol can compromise the sensory perception. However, the existence of xylitol in chewing gum formulations has been recommended in several studies to reduce plaque and inhibit mutans streptococci (Birkhed 1994; Holgerson et al. 2007; Söderling and Pienihäkkinen 2022; Söderling 2009). Thus, decreasing xylitol concentration and adding other polyols help increase the sensory preference of xylitol‐containing chewing gum samples.

Overall, the sensory evaluation results were in correlation with the instrumental color and texture findings. The overall likeliness of the samples demonstrated a positive correlation with the L* values (*r* = 0.673, *p* < 0.05), textural hardness (*r* = 0.710, *p* < 0.05), and fracturability (*r* = 0.705, *p* < 0.05). In addition, the instrumental and sensory hardness scores were correlated (*r* = 0.912, *p* < 0.05). Thus, it can be argued that instrumental hardness and fracturability values can be utilized in the estimation of sensory acceptability. It was also claimed previously that the texture of chewing gums was associated with their sensory properties (Konar et al. 2016; Palabiyik et al. 2020; Qaziyani et al. 2019).

## 4 Conclusions

In the production of sugar‐free chewing gum, the natural sweetener stevia was used instead of aspartame, and sorbitol was substituted with xylitol to investigate the effects on the color, texture, sensory properties, and stability of the chewing gums during storage as evaluated by an accelerated shelf‐life test. Stevia demonstrated no effects on the color, texture, and sensory properties of the chewing gum samples. However, xylitol caused changes in the color and texture values. The overall likeliness of the samples decreased due to xylitol.

In conclusion, stevia has high potential for use in chewing gum production. However, it should be added that this study focused on the effects of the substitution of aspartame with stevia and sorbitol with xylitol on the color, texture, and sensory properties. New studies focusing on the economical evaluation of stevia usage, its health effects, changing the concentrations and types of other chewing gum ingredients and their interactions, and changes in other quality parameters are needed. In addition, a relatively small sensory panel size was used in this study, and consumer tests with many panelists would be needed before the launch of a similar product.

## Author Contributions

**Ayse Aykut:** conceptualization (equal), data curation (equal), formal analysis (equal), investigation (equal), writing – original draft (equal). **Celale Kirkin:** conceptualization (equal), methodology (equal), supervision (equal), writing – original draft (equal), writing – review and editing (equal).

## Ethics Statement

The authors confirm that they used appropriate protocols to protect the rights and privacy of the panelists during the sensory analyses. The panelists were able to withdraw from the study at any time without reason. No personal data were collected from the participants. All tested samples were suitable for human consumption.

## Consent

A verbal consent of the panelists was obtained before the sensory analysis.

## Conflicts of Interest

The authors declare no conflicts of interest.

## Data Availability

The data that support the findings of this study are available on request from the corresponding author.
